
JOURNAL INFORMATION
==============================
NLM Title Abbreviation: Brain Pathol
Journal ID: Brain Pathol
NLM Journal ID: 9216781
Journal ID: BPA

Brain Pathology

ISSN: 1015-6305
EISSN: 1750-3639
Publisher: Wiley

ARTICLE INFORMATION
==============================
PMCID: PMC11483583
DOI: 10.1111/bpa.13308
Article ID: BPA13308
Article version: 1
Subjects: Society News, Society News

Society News

Electronic publication date: 2024 Oct 16
Publication date: 2024 Nov
Volume: 34
Issue: 6
Electronic Location ID: e13308
Copyright: © 2024 The Authors. Brain Pathology published by John Wiley & Sons Ltd on behalf of International Society of Neuropathology.
License: This is an open access article under the terms of the http://creativecommons.org/licenses/by/4.0/ License, which permits use, distribution and reproduction in any medium, provided the original work is properly cited.
License URL: https://creativecommons.org/licenses/by/4.0/

Figure count: 0
Table count: 0
Page count: 4

source-schema-version-number: 2.0
cover-date: November 2024
details-of-publishers-convertor: Converter:WILEY_ML3GV2_TO_JATSPMC version:6.4.9 mode:remove_FC converted:16.10.2024

==============================

### SOCIETY NEWS

Edited by Audrey Rousseau

**The ISN is looking for a group of young motivated neuropathologists** to promote the specialty via the ISN website. If you are interested in participating, please contact Audrey Rousseau (aurousseau@chu-angers.fr) or Monika Hofer (Monika.Hofer@ouh.nhs.uk).

**The 29th Annual Meeting and Education Day of the Society for Neuro-Oncology** will be held in November 21-24, 2024, in Houston, Texas, USA.

**The International Congress of Neuropathology (ICN)** will be held in Edinburgh, Scotland, in 2027 (ICN27). The Congress President will be Prof Colin Smith and the ICN27 will be hosted by the British Neuropathological Society (BNS).

"On behalf of the British Neuropathological Society I am delighted to extend a warm invitation to all our colleagues across the world to join us in Edinburgh for the International Congress of Neuropathology 2027. Edinburgh is an easily accessible centre, surrounded by 1000 years of living history. We will develop a strong academic programme covering all aspects of neuropathology with world leading plenary speakers, supported by a social programme highlighting some of Edinburgh's historic charms. For those wishing to explore further, Edinburgh offers access to many of Scotland's highlights, be it touring the Highlands, sampling our famous whisky or golfing on some of our picturesque courses. I do hope you will be able to join us for what I am sure will be a memorable meeting showcasing the best in international neuropathology.

Colin Smith
Congress President ISN 2027"

Summary report for ICN23 Berlin (our most recent International Congress of Neuropathology, September 2023) now available in the Society's journal Brain Pathology (see link: https://doi.org/10.1111/bpa.13249).

We are delighted to start the bidding process for holding the **2031 XXII International Congress of Neuropathology (ICN)**. As you know, the 2027 XXI ICN Congress will be in Edinburgh and we now need to think ahead to 2031 and find a new home for our much-loved congress.

The Invitation Letter calling for bids and outlining the process can be found on the ISN website (www.intsocneuropathol.com). Please note that the deadline is the **1st March 2025**.

***Brain Pathology has joined Wiley's Open Access*** portfolio as of January 2021. As a result, all submissions are subject to an Article Publication Charge (APC) if accepted and published in the journal. ISN members are eligible for a 10% discount off the Open Access APC. For more information on the fees, please click here.

***Free resource: digital microscopy platform for neurodegenerative diseases curated in Munich.*** Prof Jochen Herms and his team have been setting up a digital microscopy platform for neurodegenerative diseases in their department in Munich. Registration is free. ISN members and interested colleagues are invited to use this resource, which will be particularly useful for teaching and training (see link below). Colleagues are invited to contribute suitable cases of uncommon neurodegenerative diseases, if they wish to. The site allows photos to be taken via 'screen save' for certain characteristic pathologies (at x20) without copyright restrictions, which may be very helpful.

Link: https://znp.smartzoom.com/S6

For information on how to contribute cases please contact Prof Jochen Herms (Jochen.herms@med.uni-muenchen.de).

***ISN travel grants for trainees to visit centres of excellence.*** The ISN will annually award up to 3 grants, each of up to €1200 (approximately $1600 US), to support visits of neuropathology trainees in developing countries to neuropathology centres of excellence. The primary aim of such visits should be to provide training for the grant recipient and to promote future educational interactions between the host department and that of the trainee. The application, with a brief explanation of the reasons

THE INTERNATIONAL SOCIETY OF NEUROPATHOLOGY

**President**
Dr David Louis
Mass General Brigham and Harvard Medical School
Department of Pathology
55 Fruit Street
Boston MA 02114
USA
Email: dlouis@mgh.harvard.edu

**Past President**
Dr Seth Love
South West Dementia Brain Bank
University of Bristol
Level 2, Learning & Research
Southmead Hospital
Bristol BS10 5NB
UK
Email: seth.love@bristol.ac.uk

**Vice-Presidents**
Dr David Capper (Berlin, Germany), Email: david.capper@charite.de
Dr Catriona McLean (Melbourne, Australia), Email: c.mclean@alfred.org.au
Dr Mark Del Bigio (Manitoba, Canada), Email: marc.delbigio@umanitoba.ca

**Secretary-General**
Dr Monika Hofer
Neuropathology and Ocular Pathology Department
Level 1, West Wing
John Radcliffe Hospital
Oxford OX3 9DU
United Kingdom
Email: monika.hofer@ouh.nhs.uk

**Project Secretary**
Dr Audrey Rousseau
Departement de Pathologie Cellulaire et Tissulaire
PBH
CHU ANGERS
4, rue Larrey
49100 ANGERS
France
Email: aurousseau@chu-angers.fr

**Editor of Brain Pathology**
Dr Markus Glatzel,
Institute of Neuropathology
Universitätsklinikum Hamburg-Eppendorf
Martinistr. 52
20246 Hamburg
Germany
Email: m.glatzel@uke.de

**Treasurer**
Dr Stephan Frank
Pathologie Universitätsspital Basel
Schönbeinstrasse 40
CH-4031 Basel
Switzerland
Email: stephan.frank@usb.ch

**Archivist**
Dr Maria Thom
Department of Neuropathology
Institute of Neurology
Queen Square
London WC1N 3BG
UK
Email: m.thom@ion.ucl.ac.uk

**Chair Training and Development Subcommittee**
Dr Raj Kalaria (Newcastle, UK), Email: r.n.kalaria@newcastle.ac.uk

**Book Editors**
Dr Herbert Budka (Vienna, Austria), Email: herbert.budka@meduniwien.ac.at
Dr Homa Adle-Biassette (Paris, France), Email: homa.adle@inserm.fr

**Webmaster**
Dr David Hilton (Plymouth, UK), Email: davidhilton@nhs.net

**Ordinary members of the Executive Committee**
Dr Gabor Kovacs Marc Del Bigio (Toronto, Canada), Email: gabor.kovacs@uhnresearch.ca
*Dr Kathy Keyvani (Essen, Germany), Email: kathy.keyvani@uk-essen.de
Dr Takashi Komori (Tokyo, Japan), Email: komori-tk@igakuken.or.jp
Dr Keyvani also convenes the ISN EDI group.

© 2024 The Authors. *Brain Pathology* published by John Wiley & Sons Ltd on behalf of International Society of Neuropathology.

for the visit and intended benefits, should be made by the trainee's head of department if applicable, or another senior member of staff in his/her institution, and should be accompanied by the applicant's CV. In addition a letter of support should be sent by the head of the neuropathology department in the host institution. The application, CV and letter of support should be emailed to the Secretary General of the ISN, Monika Hofer (Monika.Hofer@ouh.nhs.uk). Applications may be made at any time and awards will be made available through the host institution.

***Bursaries to attend educational meetings.*** The ISN will provide up to 4 awards annually (and a maximum of 2 per meeting), each of up to €2500 (approximately $3400 US), to support trainees in neuropathology to attend internationally recognized courses in neuropathology, such as the Euro CNS courses (http://www.euro-cns.org/events/cme-training-courses). Please note that these bursaries are not available for attendance at the International Summer School for Neuropathology and Epilepsy Surgery, for which there is a separate awards system which should be applied for directly via the course organisers (http://www.epilepsie-register.de). Applicants should be from low-middle income, non-European/North American countries (http://data.worldbank.org/news/new-country-classifications). The application, giving a brief explanation of intended benefits from attending the course, should be made by the trainee's head of department and should be accompanied by the applicant's CV. The application, CV and letter of support should be emailed to the Secretary General of the ISN, Monika Hofer (Monika.Hofer@ouh.nhs.uk). Applications may be made at any time and the funds may be used to support registration fees, economy travel and accommodation.

***ISN newly redesigned and revised Website.*** The Society has a website (http://www.intsocneuropathol.com/) where you can find details of recent and forthcoming ISN events, publications, Society officers and links to ***Brain Pathology*** 'Under Your Microscope' cases. To access minutes of recent Council and Executive meetings, or the contact details of members, you will need to register using the tab at the bottom of the page after obtaining an invitation code from one of your councillors. We would welcome any feedback on the site and suggestions for its further use. Please send your comments to Monika.Hofer@ouh.nhs.uk.

Updates of the online Membership Directory of the ISN and of the Society News. With the move to open access for the ISN journal Brain Pathology the ISN no longer offers individual memberships and automatic ISN membership is included by signing up to individual national societies (as listed below). Any changes of contact details should be submitted to the individual national societies, who maintain their respective membership directories. Changes of the officers of National Societies and special events in these Societies should be announced by e-mail to the Project Secretary, Dr Audrey Rousseau. The Project Secretary should regularly collect information on the number of neuropathologists and neuropathology trainees in all ISN member countries. (The corresponding e-mail addresses are indicated next to the "Dear Reader" column in this issue of ***Brain Pathology***).

### NATIONAL SOCIETIES

***Argentina.*** The officers of the Argentine Society are: Dra. Ana Lıa Taratuto (ataratuto@fleni.org.ar) Department of Neuropathology, Instituto de Investigaciones Neurologicas "Dr Raul Carrea"—FLENI, and Dr Gustavo Sevlever (gsevlever@fleni.org.ar), Montanese 2325-(1428) Buenos Aires, Argentina. Tel: 154-1 788-3444; Fax 154-1 784-7620. The address of the Argentine Society is: c/o Dra. Ana Lıa Taratuto.

***Austria.*** The President of the Austrian Society of Neuropathology is is Romana Höftberger, MD, Division of Neuropathology and Neurochemistry, Department of Neurology, Medical University of Vienna, Email: romana.hoeftberger@meduniwien.ac.at. Secretary is Serge Weis, MD, PhD, Division of Neuropathology, Neuromed Campus, Kepler University Hospital, Linz, Austria, Email: serge.weis@kepleruniklinikum.at. Treasurer: Johannes Haybaeck, MD, Tyrolpath, Zams, Tirol Austria, Email: johannes.Haybaeck@tyrolpath.at. Councilors: Christine Haberler, MD, Division of Neuropathology and Neurochemistry, Department of Neurology, Medical University of Vienna, Email: Christine.Haberler@meduniwien.ac.at and Johannes Hainfellner, MD, Division of Neuropathology and Neurochemistry, Department of Neurology, Medical University of Vienna, Email: Johannes.Hainfellner@meduniwien.ac.at The address of the Austrian Society is: Österreichische Gesellschaft für Neuropathologie, c/o Dr. Romana Höftberger, Division of Neuropathology and Neurochemistry, Medical university campus/AKH 4J, Währinger Gürtel 18-20, A-1090 Vienna, Austria. Website: http://www.oegnp.at/de/.

***Australia and New Zealand.*** The president of Australian and New Zealand Society for Neuropathology is Dr. Fouzia Ziad and the secretary/treasurer is Dr. Laveniya Satgunaseelan. The councillors are Dr Michael Buckland and Dr. Thomas Robertson. The new address of the Society is: Department of Anatomical Pathology, Alfred Health, Prahran, Victoria, Australia 3181. Website: www.anzsnp.org.au.

***Baltic Association of Neuropathologists (BAN).*** A multinational neuropathological association with colleagues from Estonia, Latvia and Lithuania. The current president is Zane Jaunmuktane (zane.jaunmuktane@inbox.lv), the vice presidents are Dr. Inga Gudinaviciene (inga.gudinaviciene@lsmuni.lt) e-mail: inga.gudinaviciene@kmu.lt, Tel: +370 37 787332; Fax: +370 37 3264 05, Department of Pathology, Lithuanian University of Health Sciences Hospital Kaunas Clinics, Eiveniu 2, LT 50009, Kaunas, Lithuania) and Dr. Andres Kulia (andreskulla@hot.ee).

***Belgium.*** The president of the Belgian Society of Neuropathology is Prof Dr Patrick Cras (patrick.cras@uantwerpen.be). The treasurer is Dr Caroline Vandenbroecke (caroline.vandenbroecke@UGent.be). The secretary is Dr Alex Michotte. The address of the Belgische Groepering voor Neuropathologie/Groupement Belge de Neuropa-thologie is: Dr Alex Michotte, Department of Pathology and Neurology, AZ Vrije Universiteit Brussel, Laarbeeklaan, 101, B-1090 Brussels, Belgium; Tel: +32-2-4775080; Fax: +32-24775085; E-mail: alex.michotte@az.vub.ac.be. Website: http://www.neuro.be/

***Brazil.*** The current delegate of Neuropathology Divison (a chapter of Brazilian Society of Pathology) is Prof. MSc. Francine Hehn de Oliveira, neuropathologist at Hospital de

© 2024 The Authors. *Brain Pathology* published by John Wiley & Sons Ltd on behalf of International Society of Neuropathology.

Clınicas de Porto Alegre, Professor of Anatomic Pathology at Federal University of Rio Grande do Sul. The new adress is: Brazilian Society of Neuropathology - Division of Neuropathology (c/o Prof. Francine Hehn de Oliveira); Topazio Street, 980 - Vila Mariana - São Paulo (SP); ZIP CODE: 04105-063. Tel: +55 (11) 5080-5298. e-mail address: fran-hehn@gmail.com.

***Canada.*** The current President of the Canadian Association of Neuropathologists is Dr. Robert Hammond, Division of Neuropathology, London Health Sciences Centre, Western University, robert.hammond@lhsc.on.ca. The Secretary-Treasurer of the CANP is Dr. Peter Schutz, Division of Neuropathology, Vancouver General Hospital, peter.schutz@vch.ca. The website is http://www.canp.ca. Current representatives to the International Society of Neuropathologists are: Dr. Robert Hammond, Dr Marc Del Bigio, and Dr. Gabor Kovacs.

***China.*** Prof Yue-Shan Piao, Department of Pathology, Xuanwu Hospital, Capital Medical University, Chang Chun Street 45, Xicheng District, Beijing 100053, China. E-mail: yueshanpiao@126.com. The other member of the ISN Council is Prof Xiaokun Qi, Second Affiliated Hospital of Anhui Medical University, Hefei City, Anhui Province, China. E-mail: bjqxk@sina.com.

***Croatia.*** The following officers of the Croatian Section of Neuropathology were elected as members of the board: President: Prof Dr Vladimir Hlavka; Honorary President: Prof Dr Nenad Grcevic; Vice President: Prof Dr Dubravka Jadro-Santel, Doc. Dr Jasna Talan-Hranilovic; Secretary: Dr Kamelija Zarkovic. The address of the President is: Department of Neuropathology, University Hospital Rebro, Kispaticeva 12, 41000 Zagreb, Croatia; Tel/Fax: 1385 01/222-706.

***Czech Republic.*** The address of the Czech Republic Society of Neuropathology is: c/o Dr M. Elleder (Secretary), Hlava Institute of Pathology, Medical Faculty, Studnickova 2, 128 00 Praha 2, Czech Republic.

***France.*** The President of the French Society of Neuropathology is Prof. Emmanuelle Uro-Coste, Pathology Department, CHU Toulouse, Toulouse (uro-coste.e@chu-toulouse.fr) and the Treasurer is Dr Susana Boluda Casas (susana.boludacasas@aphp.fr).

***Germany.*** Officers are Professor Till Acker, Giessen: President, Professor Clemens Sommer, Mainz: Past President, Professor Werner Paulus, Muenster: Past President, Christian Mawrin, Magdeburg: Secretary General. www.dgnn.de/

***Greece.*** President of the Hellenic society of Neuropathology: E. Patsouris, Professor and Chairman of Pathology; Vice President: M. Panayiota, Assistant Professor; Secretary: E. Chatzigianni, Pathologist, Treasurer: C. Panayiota, Pathologist. Address for correspondence: Prof. E. Patsouris, University of Athens, Department of Pathology, Mikras Asias 75, Athens, GR-11527 Goudi, Greece; Tel: +30-210-7462158/7462017/7462229; Fax: +30-210 7462157; E-mail: panatomy@med.uoa.gr or epatsour@meduoa.gr.

***Hungary.*** President: Dr. Tibor Hortobagyi (hortobagyi@med.unideb.hu); General Secretary: Dr. Gabor G. Kovacs (gabor.kovacs@meduniwien.ac.at); Executive Committee Members: Dr. Samuel KOMOLY, Dr. Tibor Kovacs, Dr. Peter P. Molnar; Councilors are: Tibor Hortobagyi and Gabor Kovacs.

***India.*** President: Dr. Chitra Sarkar; President Elect: Dr. Vani Santosh; Secretary: Dr. Geeta Chacko; Treasurer: Dr. Nuzhat Hussain. The address of the Section of Neuropathology of India is: c/o Dr Chitra Sarkar, Department of Neuropathology, National Institute of Mental Health & Neurosciences (NIMHANS), Bangalore 560 029, India; Tel: +91-11-26593371; Fax: +91-11-26588663 E-mail: secretariat@neuropathsociety.in; Website: https://neuropathsociety.in/.

***Iran.*** Two pathologists/neuropathologists represent the speciality. Alireza Sadeghi Pour M.D., Associate professor of pathology and Lab. Medicine, Iran University of Medical Sciences-School of Medicine, Department of Pathology and Lab. Medicine, Rasool-Akram Hospital, 1445613131 Tehran, Phone and Fax: +98 21-66525587 (sadeghipour.alireza@gmail.com). Dr. Yalda Nilipour, Associate Professor of Pathology and Lab. Medicine, Pediatric Pathology Research Center, Research Institute for Children Health, Mofid Hospital, Shahid Beheshti University of Medical Sciences, Tehran, Iran (yalnil@yahoo.com).

***Ireland.*** The President of the Irish Society of Neuropathology is Dr Catherine Keohane, Consultant Neuropathologist, Department of Pathology (Neuropathology), Cork University Hospital. E-mail: katy. keohane@gmail.com ckeohane@shb.ie, the Secretary is Dr Michael A. Farrell (E-mail: mfarrell@indigo.ie) and the Treasurer is Dr Francesca M Brett (E-mail: fmbrett@iol.ie). The INP Soc continues to meet at least twice yearly to discuss difficult and interesting cases.

***Italy.*** The President of the Society is Prof. Salvatore Monaco Alessandro Mauro, Universita degli Studi di Verona Dipartimento di Scienze Neurologiche d della Visione Sezione di Neurologia Clinica Facolta di Medicina e Chirurgia, Verona (salvatore.monaco@univr.it) Dipartimento Neuroscienze, Auxologico Italiano IRCCS, Torino, E-mail: alessandro.mauro@unito.it, the Secretary Treasurer is Prof Giovanna Cenacchi, Bologna (giovanna.cenacchi@unibo.it) Elena Pegoraro, Dipartimento Neuroscienze, Universita di Padova, Padova, E-mail: elena.pegoraro@unipd.it. The councillors are Prof. Alessandro Simonati, University of Verona, and Dr. Fabrizio Tagliavini, Istituto C. Besta, Milan. The Italian Association of Neuropathology (AINP) meets once a year on annual general meeting, jointly with Italian Association for Research on the Aging Brain. The website is: http://www.neuropathology.it.

***Japan.*** The President of the Japanese Society of Neuropathology is Professor Hitoshi Takahashi. Professor Takahashi's address is as follows: Department of Pathology, Brain Research Institute, Niigata University, 1-757 Asahimachi-tohri, Chuo-ku, Niigata 951-8585, Japan, E-mail: hitoshi@bri.niigata-u.ac.jp. The Treasurer is Professor Koichi Wakabayashi, Hirosaki University Graduate School of Medicine. The International Communication Committee of the Society now comprised the following members: Haruhiko Akiyama (Tokyo Metropolitan Institute of Medical Science; Takeshi Iwat-subo (Tokyo University Graduate School of Medicine); Hitoshi Takahashi (President, see above); Kiyomitsu Oyanagi (Shinshu University School of Medicine); Hidehiro Mizusawa (National Center Hospital, National Center of Neurology and Psychiatry); and MariYoshida (Aichi Medical University). Neuropathology, the official journal of the Society, is a bimonthly published in English. The Editor-in-Chief is Professor Toru Iwaki

© 2024 The Authors. *Brain Pathology* published by John Wiley & Sons Ltd on behalf of International Society of Neuropathology.

(Graduate School of Medical Sciences Kyushu University). The Journal welcomes submissions of original papers from non-members of the Society. TheWeb Site of the Society in English is http://www.jsnp.jp/en/index.htm. Contact Person of the Society is Dr. Akiyoshi Kakita (Department of Pathology, Brain Research Institute, Niigata University, 1-757 Asahimachi-tohri, Chuo-ku, Niigata 951-8585, Japan; E-mail: kakita@bri.niigata-u.ac.jp). The ISN councilors are Akiyoshi Kakita, Niigata University, Email: kakita@bri.niigata-u.ac.jp, Shinya Tanaka, Hokkaido University, Email: tanaka@med.hokudai.ac.jp, Rie Saito, Niigata University, Email: riesaito@bri.niigata-u.ac.jp and Asuka Araki,Shimane University, Email: asuka@med.shimane-u.ac.jp.

***Mexico.*** The officers are as follows: Dr. Marco Antonio Rodriguez-Florido, President (E-mail: mar-florido@hotmail.com); Dr. Dafne Thamara Ayala-Davila, Secretary (E-mail: daf_tham@ yahoo.com.mx); Dr. Martha Lilia Tena-Suck, Treasurer (E-mail: mltenasuck@gmail.com); Dr. Maria Adelita Vizcaino-Villalobos, Officer (E-mail: ade_pumas7@yahoo.com.mx).

***The Netherlands.*** The president of the Dutch Society of Neuropathology is Dr. W.F.A. den Dunnen, Dept. of Neuropathology, Hanzeplein 1, 9713 GZ Groningen, Nederland (Email: w.f.a.den.dunnen@umcg.nl). Secretary is Dr. T.M. Teune, Maasstad Ziekenhuis (email: teunet@maasstadziekenhuis.nl).

***Poland.*** The officers of the Polish Association of Neuropathologists are: Prof. Dr hab. Janusz Szymas, President (email: jszymas@ampat.amu.edu.pl); Doc. dr hab: Teresa Wierzba-Bobrowicz, Vice President. Official WWW page of the Polish Association of Neuropathologists is: http://snp.amu.edu.pl/. This page is prepared in Polish as well in English edition. Folia Neuropathologica is the official journal of the Polish Association of Neuropathologists and the Medical Research Center of the Polish Academy of Sciences.

***Portugal.*** The president of the Portuguese Society of Neuropathology is: Prof Mrinalini Honavar.

***Scandinavia.*** The Scandinavian Neuropathological Society supports neuropathology in the five Nordic countries, ie Denmark, Finland, Iceland, Norway and Sweden. Addresses: President: Bjarne-Winther Kristensen, Denmark (bjarne.winther.kristensen@rsyd.dk). Secretary/Treasurer: Eva Løbner Lund, Denmark (eva.loebner.lund@regionh.dk). National representatives: Maria Gardberg, Finland Bård Kronen Krossnes, Norway Eva Lindberg, Sweden. The Scandinavian Neuropathological Societys counsillor in ISN is David Scheie (E-mail: david.scheie@regionh.dk). Website: http://s-n-s.org.

***South Africa.*** Dr Dan Zaharie, neuropathologist, anatomical and forensic pathologist, represents the speciality. Department of Anatomical Pathology, Neuropathology Division, Tygerberg Hospital, NHLS and Stellenbosch University, Cape Town, South Africa, PO Box 19010, Tygerberg 7505, Cape Town, South Africa, email: sdz@sun.ac.za, Tel: 1 27 21 9384041.

***South Korea.*** The President of the Korean Neuropathology Study Group (KNSG) is Professor Sang Pyo Kim (smkim5@kmu.ac.kr), Department of Pathology Keimyung University School of Medicine, Dalseong-ro 56 Jung-gu, Daegu (Zip code: 41931) South Korea and Secretary General is professor Na Rae Kim (clara_nrk@gilhospital.com), Department of Pathology, Gachon Gil Medical Center, Namdong Daero 774, Namdong-gu, Incheon, South Korea. The Web Site of the KNSG is http://www.pathology.or.kr/html/?pmode= boardlist&MMC_pid5275 (Korean). Korean version can be translated into English through Google translation. KNSG holds bi-monthly meetings with special lectures and slide conferences.

***Spain.*** The President of the Spanish Club of Neuropathology is Prof. Miguel Angel Idoate Gastearena, Department of Pathology, School of Medicine, University Clinic of Navarra, Pamplona, Spain. Email: maidoate@unav.es. The secretary is Prof. Dr. Alberto Rabano, Fundacion CIEN, Madrid.

***Switzerland.*** The President of the Swiss Society of Neuropathology is Doron Merkler, Geneva (Doron.Merkler@unige.ch), the vice president is Regina Reimann, Zurich (regina.reimann@usz.ch). The Secretary/Treasurer is Ekkehard Hewer, Lausanne (Ekkehard.Hewer@unil.ch), The ISN councillor is: Prof Constantin Bouras (constatin.bouras@medecine.unige.ch). Website: http://www.ssn.uzh.ch.

***United Kingdom.*** The President of the British Neuropathological Society is Professor Stephen Wharton (s.wharton@sheffield.ac.uk), Sheffield Institute for Translational Neuroscience. The Vice President is Professor Tammaryn Lashley, the Secretary is Professor Roxana Carare and the Treasurer is Dr Daniel du Plessis. The address of the British Neuropathological Society is: c/o Professor Roxana Carare, Clinical Neurosciences Southampton General Hospital, Southampton, So17 1BJ; email r.o.carare@soton.ac.uk. The website is (log-in required): http://www.bns.org.uk.

***United States.*** The new officers are Caterina Giannini, Malak SJ Althgafi: President, Rebecca Folkerth: Vice President, Eric Huang: President Elect, Douglas Anthony: Vice President Elect, Eyas Hattab: Vice President for Professional Affairs, Jennifer Baccon: Secretary Treasurer, Edward Lee: Assistant Secretary-Treasurer. Councillors are: Caterina Giannini, Malak SJ Althgafi, Fausto Rodriguez, Tarik Tihan, Hannes Vogel. Website: www.neuropath.org.

### JOURNAL NEWS

Stay up-to-date with the latest research in the field; simply click 'Get New Content Alerts' in the top left corner of the journal homepage and receive email notifications when new research publishes in Brain Pathology.

© 2024 The Authors. *Brain Pathology* published by John Wiley & Sons Ltd on behalf of International Society of Neuropathology.
